# The role of EGF-EGFR signalling pathway in hepatocellular carcinoma inflammatory microenvironment

**DOI:** 10.1111/jcmm.12153

**Published:** 2013-11-25

**Authors:** Peixin Huang, Xiaojing Xu, Lingyan Wang, Bijun Zhu, Xiangdong Wang, Jingling Xia

**Affiliations:** a Liver Cancer Institute, Zhongshan Hospital, Fudan UniversityShanghai, China; b Cancer Center, Fudan UniversityShanghai, China; c Biomedical Research Center, Zhongshan Hospital, Fudan UniversityShanghai, China; d Department of Respiratory Medicine, Zhongshan Hospital, Fudan UniversityShanghai, China; e Shanghai Respiratory Research InstituteShanghai, China

**Keywords:** hepatocellular carcinoma, EGF, EGFR, proliferation, migration, CXCL5, CXCL8

## Abstract

Epidermal growth factor (EGF) and their receptor (EGFR) play an important role in the development of cancer proliferation, and metastasis, although the mechanism remains unclear. The present study aimed at investigating the role of EGF-EGFR signalling pathway in the development of human hepatocellular carcinoma (HCC) inflammatory environment. Gene profiles of inflammatory cytokines from HCC were measured. Cell bio-behaviours of HCC with low or high metastasis were detected by the live cell monitoring system. Cell proliferation was measured by CCK8. The protein level of CXCL5 and CXCL8 was measured by ELISA. The phosphorylation of PI3K, ERK, MAPK was measured by western blot. EGF significantly induced cell proliferation in HepG2 cells, but not in HCCLM3 cells. EGF prompted the cell movement in both HepG2 and HCCLM3 and regulated the production of CXCL5 and CXCL8 from HCC, which were inhibited by EGFR inhibitor, Erk inhibitor (U0126), or PI3K inhibitors (BEZ-235 and SHBM1009). HCC proliferation, metastasis and production of inflammatory cytokines were regulated *via* EGF-EGFR signal pathways. CXCL5 could interact with CXCL8, possibly by CXCR2 or the cross-talk between CXCR2 and EGFR. EGF-EGFR signaling pathway can be the potential target of therapies for HCC.

## Introduction

Hepatocellular carcinoma (HCC) is one of the most common malignancies and the third most common cause of cancer mortality worldwide 1, with a poor 5-year survival rate, below 9% 2. Rapid growth of the solid tumour is one of the main characteristics of primary liver cancer 3, associated with three-dimensional aggregation, communication, differentiation and proliferation, or functioning of HCC in solid tumour engineering 4. The Inflammatory microenvironment in liver cancer was proposed to play an important role in the orientation, modelling, and functioning of HCC, mainly including inflammatory factors and cells, or cancer cells *per se* 5,6. Leucocyte recruitment, tumour cell proliferation, or metastasis and angiogenesis might be closely related to the progression and prognosis of liver cancer 7,8.

The epidermal growth factor (EGF)-epidermal growth factor receptor (EGFR) pathway was suggested to contribute to the occurrence of inflammation and HCC 9. EGF was found to facilitate DNA synthesis, regeneration, tumour growth and progression of HCC cells, and bind with EGFR as the potential connection between inflammation and HCC and one of therapeutic opportunities 3,10. However, the mechanism by which EGF-EGFR pathway was involved in the development of inflammatory microenvironment in HCC is still unclear, although EGFR inhibitor like sorafinib was approved in clinical application 11. In addition, EGF could stimulate tumour cells to produce a variety of inflammatory factors, such as interleukin (IL)-8 (CXCL8), chemokine legend (CXCL)-12, IL-6 and IL-1, to chemo-attract tumour cells and leucocytes, such as monocytes, neutrophils, or lymphocytes, from the circulation to tumour tissues, contributing to the formation of inflammatory environment 12,13. On the other hand, recruited leucocytes or activated cancer cells could secondly release the inflammatory mediators to regulate the tumour progression 14.

The present study aimed at screening the gene profiles of inflammatory factors produced from HCCs regulated by EGF and investigating the potential mechanism involved in cancer cell growth and metastasis. We also explored the role of EGF in the regulation of HCC metastasis and inflammatory microenvironment. We found that EGF could stimulate CXCL5 production from HCC through the EGF-EGFR-phosphoinositide 3 (PI3K)-kinase-extracellular signal-regulated kinases (ERK) signalling pathway and there was an interaction between CXCL5 and CXCL8 and the cross-talk between chemokine receptor-2 (CXCR2) and EGFR.

## Materials and methods

### Reagents

Human CXCL5/ENA-78 quantikine ELISA kit (DX000), recombinant human CXCL5/ENA-78, human recombinant EGF, and anti-CXCR2/CXCL8RB were purchased from R&D (Minneapolis, MN, USA). PI3K/mTOR dual inhibitor BEZ-235 was purchased from Biovision Company (Mountain View, CA, USA). SHBM1009 (a new PI3K/mammalian target of rapamycin inhibitor) was synthesized by Fudan University. Anti-p44/42 MAPK(Erk1/2), anti–phospho-p44/42 MAPK (Erk1/2) (Thr202/Tyr204), anti-p38 MAPK, anti–phospho-p38 MAPK (Thr180/Tyr182), or ERK1/2 inhibitor U0126 were from Cell Signaling Technology (Boston, MA, USA). CXCR2 inhibitor SB225002 was obtained from Calbiochem (Darmstadt, Germany).

### Cell lines and cell culture

Human HCC lines with high metastatic capacity (HCCLM3), which were established by our institute 15, with low metastatic capacity (HepG2) or normal human liver cell lines (L02) from ATCC cell bank were grown in Dulbecco's modified Eagle's medium or RPMI-1640 supplemented with 10% foetal bovine serum (FBS, Hyclone) at 37°C in a 5% CO_2_, 95% air environment in humidified incubators.

### Alive measurement of cell bio-behaviours

The cell morphological features, proliferation, differentiation, death and migration were examined by Cell-IQ (Chip-man, Tampere, Finland). About 2 × 10^5^ cells per well were plated on 24-well plates and incubated for 24 hrs. After then, cells were treated with either BEZ-235 at 1 μM (a PI3K/mTOR dual inhibitor) or SHBM1009 at 1 μM (a new PI3K/mammalian target of rapamycin inhibitor from Shanghai BioMed Co, Shanghai, China) under the stimulation of EGF at 50 or 100 ng/ml (human recombinant EGF from R&D Systems China Ltd., Shanghai, China) respectively. Each group had a blank control. The plates were transferred to Cell-IQ incubator with a special cell-secure lid after the treatment, and all-in-focus imaging recorded cells at 30-min. intervals for 48 hrs. Analysis was carried out with a freely distributed Image software (McMaster Biophotonics Facility, Hamilton, ON, Canada), by using the Manual Tracking plugin created by Fabrice Cordeliéres (Institut Curie, Orsay, France). All assays were performed in triplicate and repeated thrice. The cell morphological features, proliferation, death and migration were also tested by Leica live cell microscope DMI6000B manufactured by Leica Microsystems (Leica, Wetzlar, Germany). About 4 × 10^5^ cells per well were plated on 6-well plates. After 24 hrs, cells were treated with BEZ-235 at 1 μM) or SHBM1009 at 1 μM under the stimulation of EGF at 100 ng/ml. After the treatment, the plate was immediately transferred to Leica incubator and fluorescence imaging was taken at 30 min. intervals for 48 hrs.

### Measurement of cell proliferation

Cell proliferation was measured by using Cell Counting Kit-8 purchased from Dojindo Technologies (Kumamoto, Japan), according to the manufacture's protocol. A volume of 100 μl of suspension (3000 cells/well) was inoculated in a 96-well plate for 24 hrs. Cells were then treated with EGF at 50 or 100 ng/ml alone, or with BEZ235 at 1 μM or SHBM1009 at 1 μM for 0, 12, 24, 36 and 48 hrs. Ten microlitre of the CCK-8 solution was added to each well and incubated for 2 hrs. The absorbance at 450 nm with a reference wavelength of 630 nm of the cells was measured with a microplate reader. The wells were done in triplicate and the experiment was repeated thrice.

### Mapping of inflammatory genes

Expression of inflammatory genes was evaluated with the human RT^2^*Profiler* PCR Inflammatory Cytokines and Receptors Array (catalogue number: PAHS-011, SABiosciences, Hilden, Germany). Total RNA was isolated by using TRIZOL™ LS reagent (Invitrogen, Carlsbad, CA, USA). Two micrograms of RNA were used for cDNA synthesis with the RT^2^ First Strand Kit (SABiosciences). The RT^2^*Profiler* array was probed according to the manufacturer's protocol by using the Profiler PCR Array System and SYBR Green/Fluorescein qPCR Master Mix (SABiosciences) in an ABI 7900 sequence analyser (Applied Biosystems, Carlsbad, CA, USA). Gene expression was compared with the dedicated Web-based software package (http://www.superarray.com/pcr/arrayanalysis.php), which automatically performs all 2^−ΔCt^ based fold-change calculations from the specific uploaded raw threshold cycle data.

### Measurements of CXCL5 and CXCL8 production

Levels of CXCL5 and CXCL8 proteins in the supernatant of cell culture were determined using ELISA kits in accordance with the protocol provided by the manufacturer. Briefly, samples and standards were added in a 96-well polystyrene microplate coated with CXCL5 or CXCL8 primary antibody and incubated for 2 hrs, the plates were washed and CXCL5 or CXCL8 conjugate antibody was added and incubated for 2 hrs. After a further wash, substrate solution was added for colour development, and the reaction was terminated with stop solution. Absorbance was measured at 450 nm.

### Western blot

Protein samples (50 μg) were mixed with 1/4 volume of SDS sample buffer, boiled for 5 min., and then separated through 10% SDS-PAGE gels. After electrophoresis, proteins were transferred to nylon membranes by electrophoretic transfer. Membranes were blocked in 5% bovine serum albumin for 1 hr, rinsed and incubated with primary antibodies in TBS diluted at 1:1000 at 4°C overnight. Primary antibody was then removed by washing in TBS-tween thrice, and labelled by incubating with 0.1 mg/ml peroxidase-labelled secondary antibodies against the mouse and rabbit for 1 hr. Bands were visualized by electrochemiluminescence (ECL) and exposed to X-ray film following washing thrice in TBS-tween.

### Statistical analysis

All data were expressed as mean ± SEM. Differential values of genes were identified by using analysis of variance and/or Student's *t*-test and a fold-change cut-off was defined as the twofold above controls. Values between groups were compared by the Student's *t*-test, after anova analyses. Increased rate of total cell number was calculated as follows: Rate (%) = (value at each time-point − value of primary seeding cells)/value of primary seeding cells × 100. All experiments were repeated at least thrice and *P* < 0.05 was considered statistically significant.

## Results

**Fig 1:**
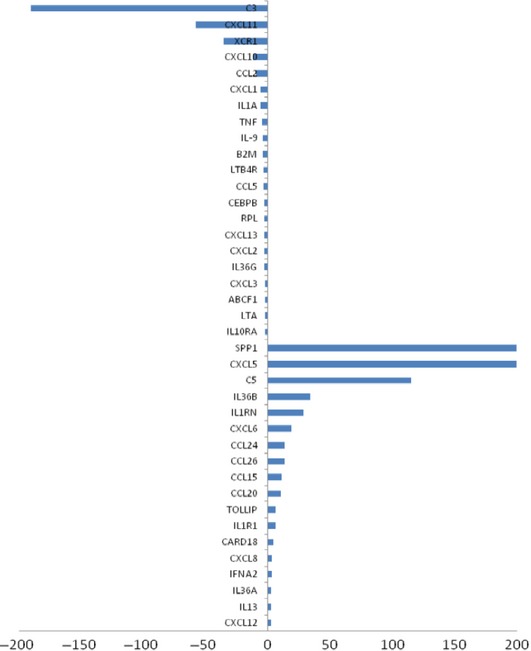
qRT-PCR array of inflammatory factors & Receptors (HCCLM3/HepG2). Inflammatory factors & Receptors with fold change >2 are shown, among which the fold change of CXCL5 and SPP1 were 1172.605 and 12,470.45 respectively (as shown in Table1).

Gene profiles of 89 inflammatory factors/receptors were screened in HCCLM3 cells with high movement capacity, HepG2 cells with low movement capacity and L02 cells as normal adult liver cells. Of them, 18 factors were up-regulated in HCCLM3, while 21 were down-regulated, as compared with HepG2 cells, as listed in Figure 1. There was a significant increased expression of CXCL5 and CXCL8 in HCCLM3 as compared with HepG2 or L02, while no statistical difference between HepG2 cells and L02, even though the expression of CXCL5 and CXCL8 in HepG2 cells was relatively high. Furthermore, we compared the expression of 89 inflammatory factors/receptors in HCCLM3 cells with or without EGF stimulation, and found that EGF induced the overexpression of seven factors/receptors, *e.g*. CXCL5, XCR1 and CXCL8, while down-regulation of 15, *e.g*. CXCL12, CCL11 and CCR3, as shown in Table 1. In addition, EGF induced up-expression of CXCL5 and CXCL8 in both HepG2 and HCCLM3 cells, as compared with L02 cells. CXCL5 and CXCL8 were selected from the screening for the further evaluation in the present study.

**Table 1:** qRT-PCR array of inflammatory factors/receptors after EGF stimulation. Inflammatory factors & receptors with fold change >2 are shown

Up-regulation	
Gene	Fold change
CCL19	2.25
CCL20	2.06
**CXCL5**	**2.01**
IL10	3.56
**CXCL8**	**2.30**
TNF	2.75
XCR1	12.40
Down-regulation	
Gene	Fold change
CCL2	1.90
CX3CR1	1.95
CCL17	1.98
CCR2	2.00
CXCL10	2.04
CCL7	2.41
CCL25	2.45
CXCL12	2.54
IFNA2	2.69
IL36RN	2.92
IL13	3.15
CCL11	3.33
CCL8	3.71
LTA	6.86
CCR3	13.50

Bold remarked factors are the target factors.

**Fig 2:**
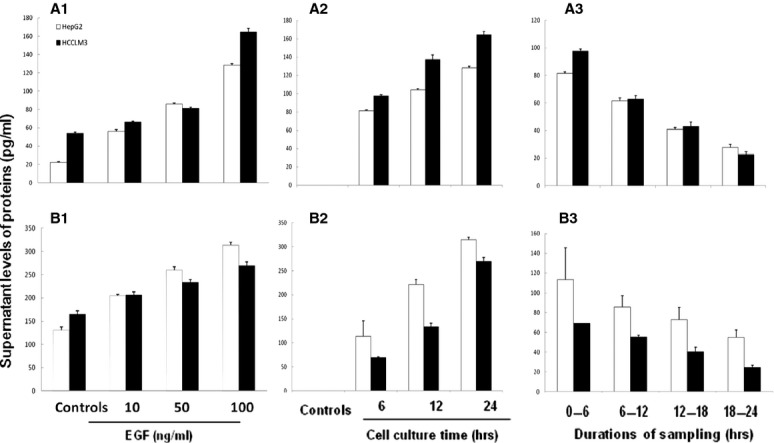
Effects of epidermal growth factor (EGF) on the production of CXCL5 and CXCL8 in hepatocellular carcinomas (HCCs). Protein levels of CXCL5 (A) and CXCL8 (B) in the supernatant harvested from HCC challenged with PBS (Controls) or EGF at 10, 50 or 100 ng/ml for 24 hrs (A1 and B1), 6, 12 and 24 hrs after EGF stimulation at 100 ng/ml (A2 and B2), or during each 6-hr period (*e.g*. 0–6, 6–12, 12–18 and 18–24 hrs) after EGF stimulation at 100 ng/ml (A3 and B3). During every 6-hour period, the full supernatant was harvested to evaluate every 6-hour production of CXCL5 and CXCL8 and the same volume of fresh solution was added.

Dose-, time-, and duration-related effects of EGF on CXCL5 and CXCL8 production were evaluated in HCCLM3 and HepG2 cells 24 hrs after the stimulation with EGF at 10, 50, or 100 ng/ml. Figure 2 demonstrated that levels of CXCL5 and CXCL8 in the supernatant significantly increased in a dose-dependent (A1 and B1) and time-dependent pattern (A2 and B2; *P* < 0.05 or less respectively). The peak productions of CXCL5 and CXCL8 occurred at the first 6 hrs after EGF stimulation (A3 and B3). Six-hour effects of EGF on CXCL5 or CXCL8 production from HCCs were evaluated by measuring CXCL5 or CXCL8 concentrations every 6 hrs after the culture medium was fully replaced with the fresh medium with EGF at 100 ng/ml. Levels of CXCL5 in HCCLM3 were significantly higher than in HepG2 stimulated with EGF at 100 ng/ml (Fig. 2A1), or from 6 hrs and onwards after EGF stimulation (Fig. 2A2) and at the first 6 hrs after EGF stimulation at 100 ng/ml (Fig. 2A3) respectively (*P* < 0.05 or less). Levels of CXCL8 in HepG2 were significantly higher in HCCLM3 stimulated with EGF at 50 and 100 ng/ml (Fig. 2B1), from 6 hrs and onwards after EGF stimulation (Fig. 2B2), and during each 6-hr period after EGF stimulation at 100 ng/ml (Fig. 2B3) respectively (*P* < 0.05 or less). Levels of CXCL5 and CXCL8 in the supernatant harvested from HepG2 cells stimulated by EGF at 100 ng/ml were significantly higher than those from HCCLM3 stimulated with vehicle (*P* < 0.05 or less, respectively, Fig. 2A1 and B1).

**Fig 3:**
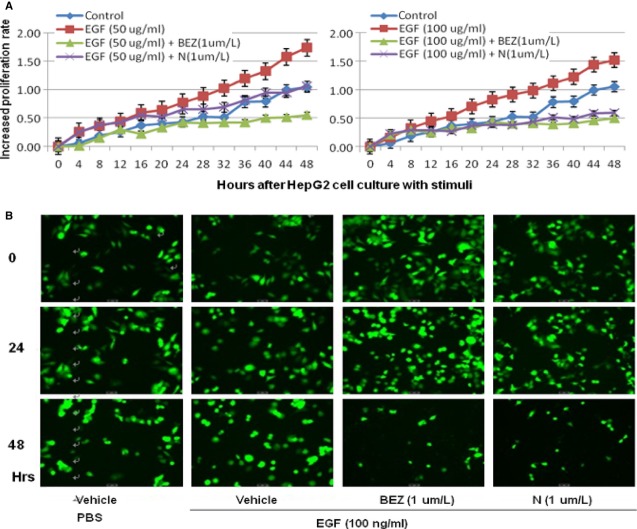
Bio-behaviours of hepatocellular cancer cells. (HepG2). Dynamic alterations in increased rate (A) of total HepG2 cells with the loading cell number at the corresponding group measured by the real-time cell monitoring system at each 30 min. for 48 hrs after a 24-hr culture of HepG2 pre-treated with vehicle (Control), BEZ235 (BEZ), or SHBM1009 (N) for 1 hr followed by the challenge with PBS or epidermal growth factor at the dose of 50 or 100 ng/ml respectively. Images of HepG2 cells (B) before the termination of experiment (×100).

**Fig 4:**
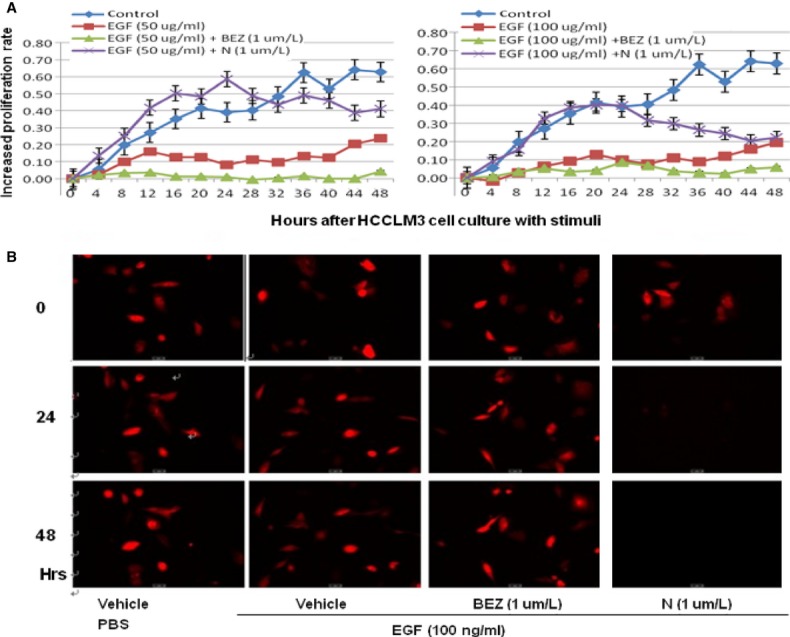
Bio-behaviours of hepatocellular cancer cells (HCCLM3). Dynamic alterations in increased rate (A) of total HCCLM3 cells with the loading cell number at the corresponding group measured by the real-time cell monitoring system at each 30 min. for 48 hrs after a 24-hr culture of HepG2 pre-treated with vehicle (Control), BEZ235 (BEZ), or SHBM1009 (N) for 1 hr followed by the challenge with PBS or epidermal growth factor at the dose of 50 or 100 ng/ml respectively. Images of HCCLM3 cells (B) before the termination of experiment (×100).

The proliferation rate of the total cells significantly increased in HepG2 (Fig. 3) and HCCLM3 cells (Fig. 4) by time after the stimulation with EGF at 50 and 100 ng/ml, between which the rate of HepG2 was higher than HCCLM3 under EGF stimulation. Treatment with BEZ235 significantly prevented increased proliferation rate induced by EGF at both 50 and 100 ng/ml, while SHBM1009 showed inhibitory effects at 100 ng/ml (*P* < 0.05, respectively, Fig. 3). The percentage of total HCCLM3 cells significantly increased by time in cells treated and stimulated with vehicle, as compared with cells treated with vehicle or BEZ235 and stimulated with EGF at 50 or 100 ng/ml (*P* < 0.05 or less, Fig. 4A). The number of HCCLM3 cells treated with SHBM1009 increased at the first 24 hrs after the stimulation with EGF, while decreased at the late 24 hrs (Fig. 4), of which the levels were significantly lower than those in Controls, but still higher than cells treated with vehicle or BEZ235 and stimulated with EGF (*P* < 0.05). The number of cells with BEZ235 and EGF was significantly lower than those with vehicle and EGF. Those findings were further demonstrated by the imaging and account of HepG2 or HCCLM3 cells labelled with fluorescence in Figures 3B and 4B.

**Fig 5:**
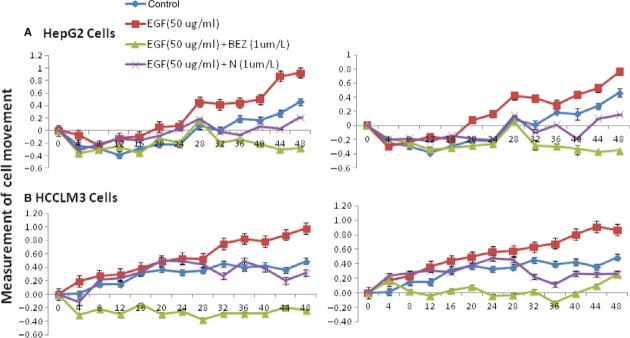
Effect of epidermal growth factor (EGF) on hepatocellular carcinoma's movement. Dynamic alterations in the movement of HepG2 (A) and HCCLM3 cells (B) with the loading cell number at the corresponding group measured by the real-time cell monitoring system at each 30 min. for 48 hrs after a 24-hr culture of HepG2 pre-treated with vehicle (Control), BEZ235 (BEZ), or SHBM1009 (N) for 1 hr followed by the challenge with PBS or EGF at the dose of 50 or 100 ng/ml respectively.

Figure 5 described that the movement of HepG2 (A) and HCCLM3 (B) treated with vehicle significantly increased after the stimulation with EGF at 50 or 100 ng/ml, as compared with those treated with vehicle, BEZ235 or SHBM1009 (*P* < 0.05 or less respectively). The movement of cells treated with BEZ235 was significantly lower than those with vehicle or SHBM1009 (Fig. 5). Proliferation of HepG2 cells stimulated with EGF significantly increased at 24 hrs, which was significantly inhibited by the treatment with Erlotinib (EGFR inhibitor) at 1 and 10 μM, U0126 (ERK inhibitor) at 5 and 10 μM, BEZ235 at 1 μM, or SHBM1009 at 1 μM, as compared with control (*P* < 0.05 or 0.01, respectively, Fig. S1A). Proliferation of HCCLM3 cells treated with vehicle, Erlotinib, or U0126 significantly increased 24 hrs after the stimulation with EGF, but not those treated with BEZ235 or SHBM1009, as shown in Figure S1B.

**Fig 6:**
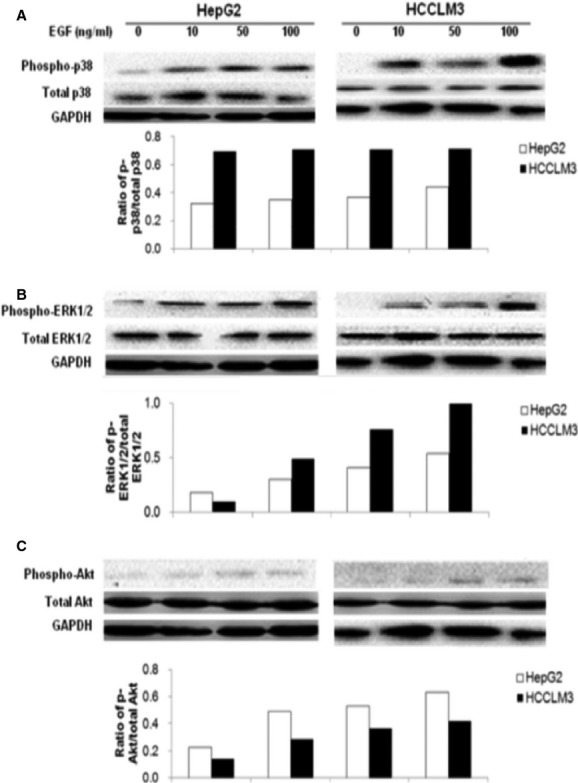
Activation of ERK1/2, p38and PI3K/Akt induced by epidermal growth factor (EGF). The levels of total p38 MAPK and phosphorylated p38 MAPK (phospho-p38) (A), total ERK1/2 and phosphorylated ERK1/2 (phospho-ERK1/2) (B), and total Akt and phosphorylated Akt (phospho-Akt) (C) in HepG2 and HCCLM3 measured by Western blot, the average level of three identical experiments (*n* = 3 each). Starved cells were challenged without (0) or with EGF at doses of 10, 50 and 100 ng/ml.

Figure 6 demonstrated the phosphorylation of p38 MAPK (A), ERK1/2 (B) or Akt (C) measured in HepG2 or HCCLM3 cells cultured without serum for 24 hrs and treated with EGF at 10, 50 or 100 ng/ml for 1 hr. Ratios between phosphorylated ERK1/2 and total ERK1/2 or between phosphorylated Akt and total AKT in both cells significantly increased in a dose-dependent pattern (*P* < 0.05). EGF induced higher ratios between phosphorylated ERK1/2 and total ERK1/2 and lower ratio between phosphorylated Akt and total Akt in HCCLM3 cells, as compared with those in HepG2.

**Fig 7:**
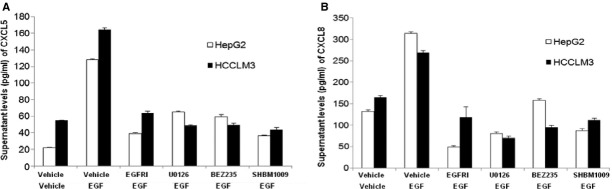
Effect of EGFR, PI3K/Akt, and ERK1/2 inhibitors on cell proliferation. Supernatant levels of CXCL5 (A) and CXCL8 (B) produced from HepG2 and HCCLM3 cells treated with Erlotinib (EGFRI), U0126, BEZ235, or SHBM1009 for 1 hr followed by the stimulation with vehicle or epidermal growth factor at 50 or 100 ng/ml for 24 hr.

**Fig 8:**
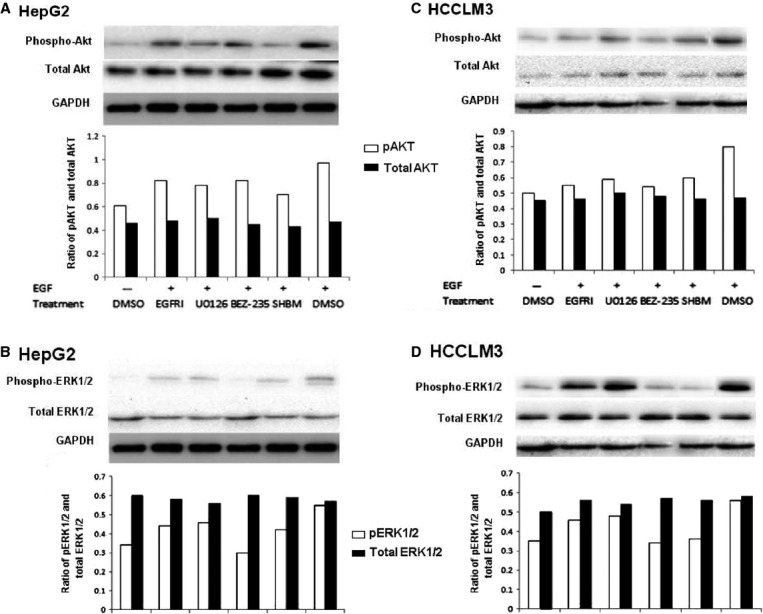
Inhibitory effects on phosphorylation of Akt and Erk. Total and phosphorylated Akt (A) and Erk1/2 (B) in HepG2 cells and total and phosphorylated Akt (C) and Erk1/2 (D) in HCCLM3 cells treated with vehicle (DMSO), erlotinib (EGFRI), U0126, BEZ235 and SHBM1009 (SHBM) and stimulated with vehicle or epidermal growth factor at 100 ng/ml.

To further evaluate the role of EFG-activated signal pathways in the production of CXCL5 and CXCL8, HepG2 or HCCLM3 cells were treated with vehicle, elrotinib, BEZ235, or SHBM1009 at 1 μM, or U0126 at 10 μM for 2 hrs, respectively, and then stimulated with EGF at 100 ng/ml for 24 hrs. Figure 7 demonstrated that levels of CXCL5 (A) and CXCL8 proteins (B) in HCCLM3 cells treated and stimulated with vehicle were significantly higher than those in HepG2 cells (*P* < 0.01). EGF stimulation significantly increased the production of CXCL5 and CXCL8 from HepG2 and HCCLM3 cells, which was prevented by the treatment with elrotinib, U0126, BEZ235 or SHBM1009 (*P* < 0.05 or less, Fig. 7A and B). Levels of CXCL5 in HCCLM3 were higher, while CXCL8 in HepG2 cells were higher than in HCCLM3 cells after EGF stimulation. Treatment with EGFRI more significantly prevented EGF-induced production of CXCL5 and CXCL8 from HepG2, while U0126 or BEZ-235 had more effective on HCCLM3. Although all inhibitors showed inhibitory effects, EGF-stimulated phosphorylation of AKT in HepG2 cells was more significantly down-regulated by the treatment with SHBM1009 (Fig. 8A), while ERK1/2 with BEZ235 (Fig. 8B). All inhibitors significantly down-regulated EGF-stimulated phosphorylation of AKT in HCCLM3 Fig. 8C), while BEZ235 or SHBM1009 was more effective on the phosphorylation of ERK1/2 (Fig. 8D).

**Fig 9:**
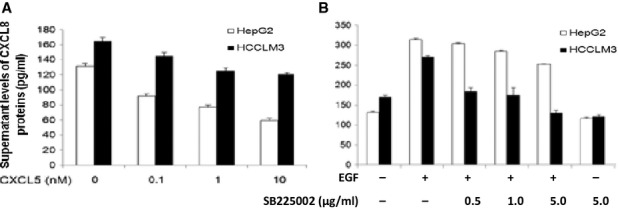
Effects of CXCL5/CXCR2 on CXCL8 production. Supernatant levels of CXCL8 proteins produced from HepG2 and HCCLM3 cells stimulated with CXCL5 at 0.1, 1.0 or 10 nM for 24 hrs (A), or treated with vehicle or CXCR2 inhibitor (SB225002) at 0.5, 1.0 or 5.0 μg/ml for 1 hr followed by the stimulation with epidermal growth factor at 100 ng/ml for 24 hrs (B).

To investigate the effect of extraneous CXCL5 on endogenous productions of CXCL8, HepG2 and HCCLM3 were incubated with CXCL5 at the dose of 10, 50 or 100 ng/ml for 24 hrs. Treatment with extraneous CXCL5 significantly inhibited the production of CXCL8 from HepG2 and HCCLM3 cells in a dose-dependent pattern (Fig. 9A). Levels of EGF-induced CXCL8 production from HCCLM3 cells were significantly lower when treated with SB225002 at 0.5–5.0 μg/ml and from HepG2 cells when Sb225002 at 5.0 μg/ml, as compared with EGF-stimulated cells treated with vehicle (*P* < 0.05 or less, Fig. 9B).

## Discussion

The development of HCC was associated with persistent and chronic liver inflammation. Inflammatory cytokines and their receptors as important components of inflammatory microenvironments play vital roles in the development and progression of HCC. EGF-EGFR signalling pathway may be one of the candidates to link between inflammation and liver cancer and act as potential therapeutic targets 16. EGF, a 6045-kD protein with 53 amino acid residues and three intra-molecular disulphide bonds, can stimulate cell growth, proliferation, survival and differentiation 16,17. EGFR, a 170 kD transmembrane glycoprotein, which is known as ErbB1, is made up of an extracellular ligand-binding domain, a single a-helical transmembrane domain, and a cytoplasmic domain that harbours a tyrosine kinase region 17–19. EGF and EGFR were overexpressed in many cancers, such as breast, lung and liver cancer 17,20,21. Our present study provides evidence that EGF plays an initial role in the development of the cancer inflammation and in the promotion of cancer cells from low metastatic potentials into high metastatic potential. Cells with low metastatic capacity were more sensitive to EGF stimulation than cells with high metastatic capacity, associated with the formation of inflammatory microenvironment and the regulation of HCC proliferation and migration.

Epidermal growth factors could be produced by autocrine and paracrine from HCCs 22 and overexpression of EGF/EGFR might be associated with poor prognosis of patients with HCC 23. EGF-EGFR banding could trigger the activity of intrinsic protein-tyrosine kinase 24, initiating a signal transduction cascade including ERK-PI3K-Akt pathway, the ras/raf/MEK/MAPK cascade and nuclear factor kappaB (NF-kB). EGF-induced biochemical changes within the cell ultimately lead to DNA synthesis and cell proliferation, cell division, wound healing, carcinogenesis and tumour progression 17,25. The continuous activation of EGF-EGFR signalling pathway is considered as a key factor in inflammation and modulated tumour proliferation, differentiation, epithelial–mesenchymal transition and angiogenesis within HCC 26–28, although the mechanism by which EGF regulates the occurrence of inflammatory microenvironment remains unclear.

The present study provides the primary evidence that EGF could regulate HCC cells to overexpress and produce mRNA and proteins of CXCL8 and CXCL5. The overexpression of CXCL5 and CXCL8 mRNA as two of mapped inflammatory factors was noticed in HCCs with high metastatic potentials, while also in HCCs with low metastatic potential after EGF stimulation. It indicates that EGF may initiate and contribute to the development of HCC inflammatory microenvironment by the production of leucocyte chemo-attractants like CXCL5 and CXCL8 from HCC *per se*, probably responsible for the movement of tumour cells and recruitment of circulating leucocytes into the certain location 29. We found that EGF could increase the proliferation of HCC cells with low metastatic potential and the movement of both HCC cells with low or high metastatic capacity. Interestingly, EGF with high concentrations presented inhibitory effects in proliferation of HepG2 cells, probably because of EGF biphasic effect that EGF in high concentrations may trigger a complicated pathway to induce cell apoptosis. Erk and Src/EGFR/STAT5 may function as a switch of EGF signalling depending on EGF concentration 30.

CXCL5 is a small cytokine belonging to the CXC chemokine family that is also known as epithelial-derived neutrophil-activating peptide 78 (ENA-78). CXCL5 has chemotactic and activating functions on neutrophil, mainly during acute inflammatory responses. However, up-expression of CXCL5 was also found in malignant tumours, such as breast cancer, nasopharyngeal cancer and also HCC, and closely correlated with poor prognosis 31–33. CXCL8 also plays an important role in the migration of leucocytes and metastasis of cancer cells in HCC microenvironment 34–36, although the function of CXCL8 needs to be further clarified. Our data indicate that HCC cells *per se* can play an important role in the development of inflammatory microenvironment through the endocrine secretion of CXCL5 and/or CXCL8. We found that up-expression of CXCL5 and IL-8 mRNA and protein in HCC might be correlated with the metastasis. There was evidence that tissue-specific regulation of CXCL8 in leucocyte recruitment depended upon monomer–dimer equilibrium and glycosaminoglycan interactions of chemokine CXCL8 37. Overexpression of CXCL8 was observed in HCC tissues, associated with the incidence of microscopic vessel invasion, pathological stages of HCCs, or potential of metastasis 38. It is also demonstrated by recent studies from our colleagues that CXCL5 mRNA and protein were overexpressed in patients with HCC and validated in animal model, associated with metastatic potentials and the development of inflammatory microenvironment through direct chemoattractant effects 39. We believe that CXCL5 and CXCL8 originated from HCC cells may be indicators of cell movement, shorter overall survival and tumour recurrence.

The EGFR pathway was proposed to serve as a ‘signalling hub’ for an increasing number of inflammatory mediators and possibly engage in extensive cross-talks with other signalling pathways 40. The present study demonstrated that EGF directly and efficiently stimulated the overproduction of CXCL8 and CXCL5 in a dose-and time-dependent manner, which was inhibited by EGFR inhibitor. It suggested that the EGF-EGFR signalling pathway plays a crucial role in mechanism of HCC-origin production of CXCL8 and CXCL5 and EGF-dominated proliferation and movement of HCC cells. Furthermore, EGF could activate EGFR-downstream signalling pathways *e.g*. PI3K or ERK pathways, in a dose-dependent manner, to mediate the inflammatory microenvironment, cell proliferation, apoptosis, migration and metastasis, as supported by other studies 16,41,42. The down-regulation of PI3K and ERK pathway by PI3K and ERK inhibitors could inhibit the cell proliferation and migration in HCC cells and the production of CXCL8 and CXCL5 from HCC cells. These results imply that the inflammatory factors produced by HCC cells could be regulated through EGF-EGFR and PI3K and ERK pathways, responsible for the formation of tumour inflammatory microenvironment.

CXCR2, the co-receptor of CXCL8 and CXCL5, initially known as a neutrophil chemokine receptor, has also been found expressed on liver cells. It was reported that overexpression of CXCR2 was correlated with intrahepatic metastasis, portal cancer embolus and tumour-lymph node-metastasis staging of HCC 43–45. It is possible that competitive bindings of CXCL5 with CXCR2 may have a negative feedback to the production of CXCL8, which helps to explain the co-regulation of CXCL5 and CXCL8. CXCR2 was proposed to be involved in the tumour malignancy *via* EGFR-dependent mechanism 46, while CXCL8 might play a potential role in tumour development by EGFR transactivation 47. Our study indicates that the cross-talk may exist between CXCR2 and EGFR in HCC cells, demonstrated by the finding that the EGF-induced production of CXCL8 was decreased by CXCR2 inhibitor in a dose-dependent pattern. The possibility is the occurrence of cross-talk between CXCR2 and EGFR transactivation. On the other hand, the finding that CXCR2 was highly expressed in HCC cells with high metastatic potential may explain why EGF could induce more production of CXCL5, while less of CXCL8, in HCC cells with high metastatic potential, and support the necessary role of CXCR2 in the production of CXCL8. Overexpression of CXCL8 was observed in HCC tissues, associated with the incidence of microscopic vessel invasion, pathological stages of HCCs or potential of metastasis 38. It would be more expected to further investigate the interaction and network between those inflammatory mediators and their receptors, between involved signal pathways, as well as between transcription factors in the formation of HCC inflammatory microenvironment using new approaches like systems biology and clinical bioinformatics 48–52.

**Fig 10:**
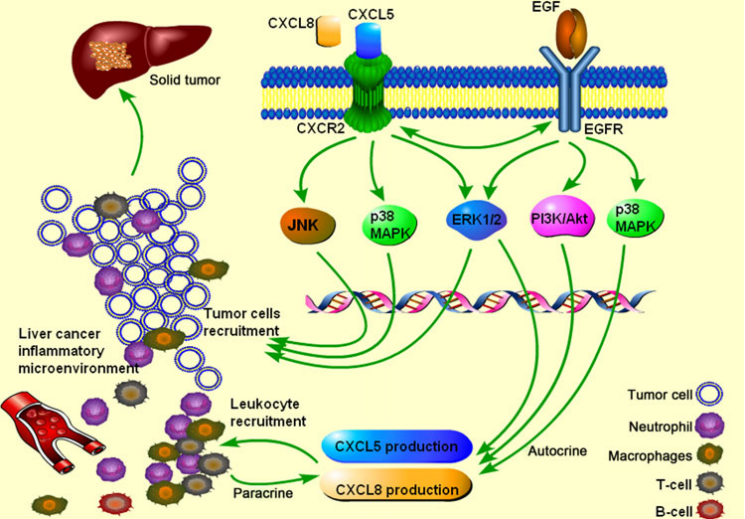
The role of epidermal growth factor (EGF-EGFR) signalling pathway in hepatocellular carcinoma (HCC) inflammatory microenvironment. The role of EGF-EGFR signalling pathway in the department of inflammatory microenvironment of HCC. HCC cells *per se* could produce inflammatory factors such as CXCL5 and CXCL8, probably responsible for the recruitment of leucocytes and HCC metastasis. EGF may act as an initiator factor to facilitate the transforming process of tumour cells from low metastatic potential into high metastatic potential, associated with the regulation of tumour progression. The potential mechanism indicated from the present study is that EGF may combine with its receptor EGFR to initiate the downstream of PI3K and Erk signal pathway and contribute to HCC proliferation, migration and production of inflammatory cytokines. HCC-produced inflammatory cytokines further form dynamic networks in tumour microenvironment and interact with each other. CXCR2 as the co-receptor of CXCL5 and CXCL8 probably have the cross-talk with EGFR. CXCL5 and IL-8 may further regulate the inflammatory microenvironment by combing with CXCR2.

In conclusion, we initially found that EGF could stimulate mRNA expression and protein production of CXCL5 and CXCL8 from HCC cells, of which HCC cells with low metastatic potential were more sensitive to EGF stimulation by overproduction of CXCL8 and proliferation. EGF could increase the movement of HCC cells, especially transforming HCC cells from low to high metastatic capacity, through EGF-EGFR binding, PI3K and ERK signal pathway, or EGFR transactivation of CXCR2. CXCL5 interact with CXCL8 possibly *via* their co-receptor CXCR2. Thus, our data indicate that EGF may be the key factor in the development of HCC inflammatory microenvironment. We hypothesize that EGF may act as an initiator factor, also called ‘first-hit factor’, to induce the occurrence of HCC inflammatory microenvironment and increase HCC metastasis, and then HCC-origin inflammatory cytokines (*e.g*. CXCL5 and CXCL8) may act as the second hit to metastasis and development of HCC (Fig. 10). EGF-EGFR signalling pathway can be the potential target of therapies for HCC inflammatory microenvironment.

## Supporting information

Figure S1 Effect of EGFR, PI3K/Akt and ERK1/2 inhibitors on cell proliferation. Cell proliferation was measured by CCK8 24 hrs after HepG2(A) or HCCLM3 (B) were pre-treated with vehicle, EGFR at 1 or 10 μM, U0126 at 5 or 10 μM, BEZ235 and SHBM1009 for 1 hr, followed by the challenge with EGF at 50 or 100 ng/ml.
