# Supplementary figures and images for: The role of EGF-EGFR signalling pathway in hepatocellular carcinoma inflammatory microenvironment
Source: J Cell Mol Med. 2013 Nov 25;18(2):218–30. doi: 10.1111/jcmm.12153 (PMC3930409; doi:10.1111/jcmm.12153)

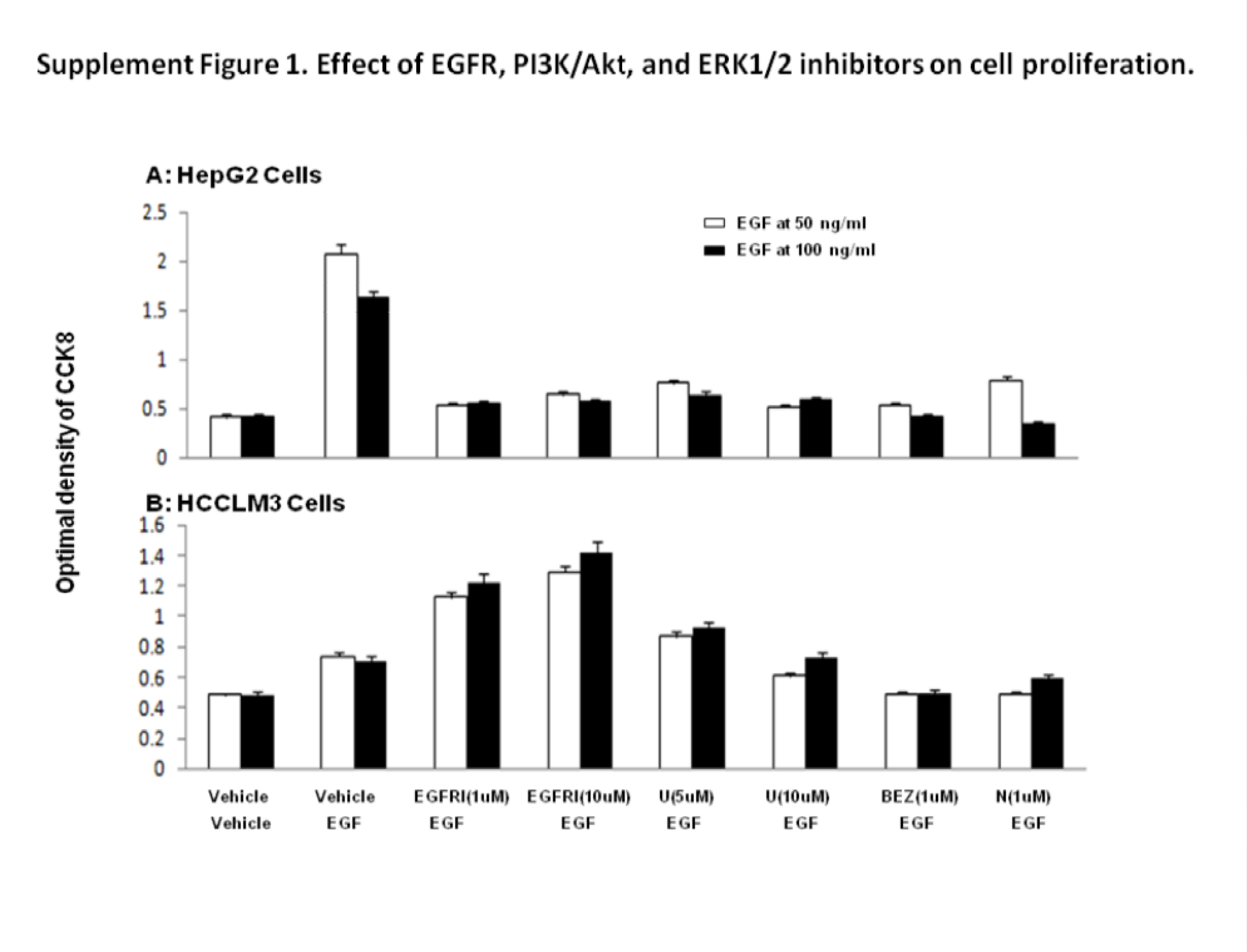

Supplement: Figure S1 — Effect of EGFR, PI3K/Akt and ERK1/2 inhibitors on cell proliferation. Cell proliferation was measured by CCK8 24 hrs after HepG2(A) or HCCLM3 (B) were pre-treated with vehicle, EGFR at 1 or 10 μM, U0126 at 5 or 10 μM, BEZ235 and SHBM1009 for 1 hr, followed by the challenge with EGF at 50 or 100 ng/ml. [file jcmm0018-0218-sd1.tif]
